# IL-19 and Other IL-20 Family Member Cytokines in Vascular Inflammatory Diseases

**DOI:** 10.3389/fimmu.2018.00700

**Published:** 2018-04-06

**Authors:** Michael V. Autieri

**Affiliations:** Department of Physiology, Independence Blue Cross Cardiovascular Research Center, Temple University School of Medicine, Philadelphia, PA, United States

**Keywords:** interleukin-19, atherosclerosis, restenosis, smooth muscle cell, interleukin-20, vascular inflammation

## Abstract

Cardiovascular disease remains a major medical and socioeconomic burden in developed and developing countries and will increase with an aging and increasingly sedentary society. Many vascular diseases and atherosclerotic vascular disease, in particular, are essentially inflammatory disorders, involving multiple cell types. Communication between these cells is initiated and sustained by a complex network of cytokines and their receptors. The interleukin (IL)-20 family members, IL-19, IL-20, IL-22, and IL-24, initiate, sustain, and drive the progression of vascular disease. They are important in vascular disease as they facilitate a bidirectional cross-talk between resident vascular cells with immune cells. These cytokines are grouped into the same family based on shared common receptor subunits and signaling pathways. This communication is varied and can result in exacerbation, attenuation, and even repair of the vasculature. We will briefly review what is known about IL-20, IL-22, and IL-24 in cardiovascular biology. Because IL-19 is the most studied member of this family in terms of its role in vascular pathophysiological processes, the major emphasis of this review will focus on the expression and atheroprotective roles of IL-19 in vascular inflammatory disease.

## Introduction

### Cytokines and Vascular Inflammation

Atherosclerotic vascular disease continues to represent a significant morbidity and mortality in westernized societies. It contributes to the severity of multiple diseases and exacerbates common comorbidities such as obesity, metabolic syndrome, and Type II diabetes. The lipid theory of atherosclerosis postulates that an excess of low-density lipoprotein is modified by oxidization, becomes lodged in the arterial subendothelial space, and acts as an antigenic, pro-inflammatory compound (1). Cytokines induce the expression of cell adhesion molecules on the surface of endothelial cells (ECs), leading to increased leukocyte extravasation. Persistent inflammation recruits additional monocytes into the subendothelial space where they differentiate into macrophages and continue to engulf oxLDL and secrete cytokines (2). Pro-inflammatory cytokines also cause vascular smooth muscle cells (VSMCs), which comprise the major cellular component of the artery, to transform from their normally differentiated, contractile phenotype to an activated “synthetic” state (3). Synthetic VSMCs migrate from the media into the intima and proliferate, phagocytize oxLDL, and secrete additional pro-inflammatory cytokines, which in turn recruit additional VSMCs as well as immune cells (4, 5). This vicious circle is sustained as long as oxidized lipid is present to drive this process. Vascular interventional procedures such as balloon angioplasty and stent placement attempt to minimize plaque occlusion, but often damage the endothelium, resulting in vascular restenosis (6). A compromised endothelial layer results in activated VSMC migration from the media into the intima of the artery, forming a “neointima” consisting primarily of proliferating VSMC (7, 8). While primarily a vascular proliferative disorder, vascular restenosis does have an inflammatory component and is driven by cytokine synthesis from VSMC and infiltrating immune cells.

The microenvironment of the atherosclerotic plaque is a dynamic collection of multiple cell types including ECs, VSMCs, and infiltrating inflammatory cells which communicate with each other through a series of cytokine-receptor-mediated interactions (9). A balance of pro- and anti-inflammatory cytokines can determine plaque severity and stability. Indeed, a recent clinical trial in which a therapeutic monoclonal antibody targeting the pro-inflammatory cytokine interleukin (IL)-1β (10) led to significantly lower rate of cardiovascular events, underscoring the importance of pro-inflammatory cytokines in the etiology and progression of vascular diseases. Cytokines are frequently associated with their effects on T helper (Th) cells, with pro-inflammatory cytokines being associated with Th1 and anti-inflammatory cytokines characterizing Th2. Th1 cytokines such as TNFα, IL-1β, and IFNγ are much more prevalent in human atherosclerotic lesions than the Th2 cytokines (11, 12). Because atherosclerotic lesions are overwhelmingly pro-inflammatory, fewer studies have pursued the characterization of anti-inflammatory, Th2-biased cytokines. Modulation of anti-inflammatory or protective cytokines could tip the balance of these “opposing forces” from a Th1, to an anti-inflammatory Th2, plaque milieu.

### The IL-20 Family Member Cytokines

**Figure 1:**
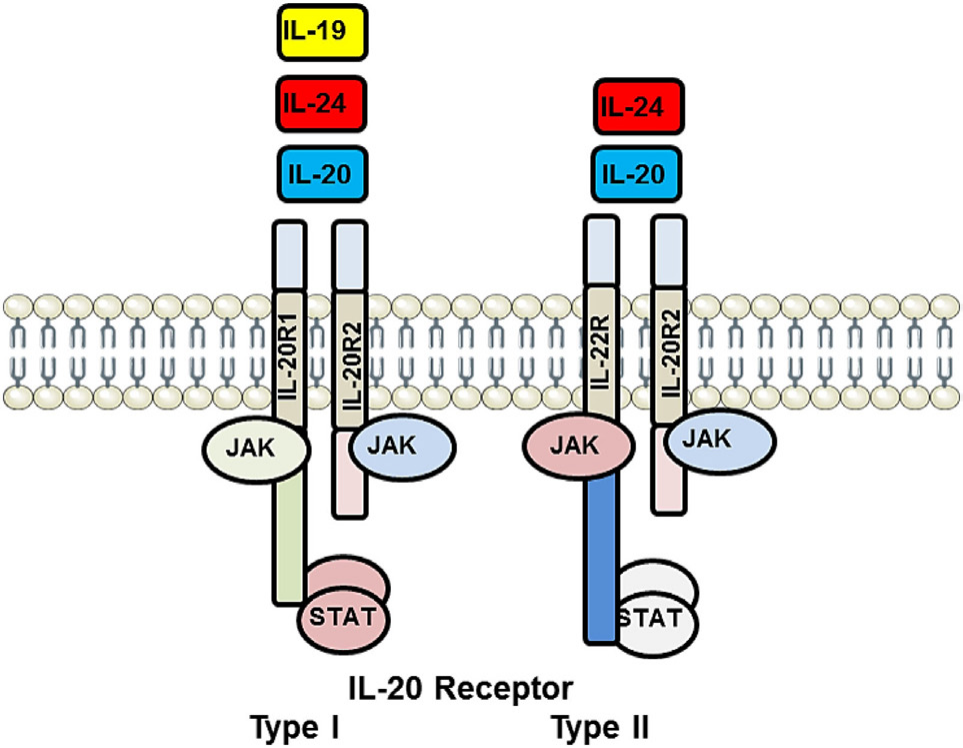
Illustration of interleukin (IL)-20 receptor system utilized by IL-19, IL-20, and IL-24. IL-20 receptor Type I is heterodimer composed of IL-20R1 and IL-20R2 and is recognized by IL-19, IL-20, and IL-24. IL-20 receptor Type II is a heterodimer composed of IL-20R2 and IL-22R and recognized only by IL-20 and IL-24. Each of these receptor complexes is capable of interacting with and activating a repertoire of JAK and STAT-signaling proteins. This multiplicity of ligand recognition and effector protein utilization offers combinational diversity in signal transduction and distal cellular processes. JAK, Janus Kinase; STAT, signal transducer and activator of transcription.

Interleukins 19, 20, 22, and 24 all signal through receptor complexes containing the IL-20 receptor β chain (IL-20Rβ) (13, 14). Three cytokines, IL-19, IL-20, and IL-24, can signal through the IL-20 Type I receptor composed of a heterodimer formed by IL-20Rα and IL-20Rβ. IL-20 and IL-24, but not IL-19, can also signal through the IL-20 Type II receptor composed of a heterodimer formed by IL-22Rα and IL-20Rβ (Figure 1). Upon engagement of their respective receptor complex, all IL-20 subfamily members activate the Janus Kinase (JAK) and signal transducer and activator of transcription (STAT) pathway, particularly STAT3 (15). These subfamily members are primarily produced by inflammatory cells, but epithelial cells, and particularly in the case of IL-19, resident vascular cells (EC and VSMC), can produce and respond to these family members. Little is reported on IL-20, IL-22, and IL-24 involvement in vascular disease; we will briefly review involvement of these family members in the development of vascular disease; the majority of this review will focus on the expression, role, and molecular mechanisms of IL-19 activity in vascular inflammatory disorders.

### Interleukin-20

Interleukin-20 is best known for the regulation of proliferation and differentiation of keratinocytes during skin inflammation (13). In terms of vascular pathology, IL-20 and its receptor subunits are detectible in macrophage-rich areas and endothelium in human and murine atherosclerotic plaque, and IL-20 expression is induced in macrophage and EC when challenged with inflammatory stimuli (16). IL-20 is not expressed in VSMC. While oxLDL induced the expression of IL-20 in monocytes, it did not affect oxLDL uptake by macrophages. Despite its structural similarity to IL-10, IL-20 is pro-inflammatory and induces chemokine and VEGF expression in EC (17). Not surprisingly, intramuscular electroporation of IL-20 cDNA into ApoE−/− mice enhanced atherosclerotic plaque area, but did not increase macrophage infiltrate into lesions in these mice (16). Because leukocyte infiltrate was not increased in these lesions, and its induction of VEGF in EC, it is suggested that IL-20 pro-atherosclerotic effects are due to increased lesion microvascularization, rather than by a pro-inflammatory effect. Indeed, one non-immune function of IL-20 appears to be the promotion of angiogenesis in hypoxic tissue and proliferation and angiogenic tube formation in cultured EC (17, 18). Thus, IL-20 involvement in atherosclerosis may be considered to be circumstantial, through its pro-angiogenic activity.

### Interleukin-22

Interleukin-22 is expressed at its highest levels in CD4+ T lymphocytes and is not reportedly expressed in non-immune cells. There is little literature which focuses on a role for this cytokine in vascular disease, but based on one report, IL-22 may play a protagonistic role in vascular inflammatory disease. IL-22/ApoE double knockout mice fed a high-fat diet developed significantly smaller lesions compared with ApoE controls (19). This study also found that IL-22-deficient mice had higher levels of SMC contractile gene expression, perhaps reflecting less local inflammation. However, IL-22 deficiency did not affect the release of pro- and anti-inflammatory mediators in immune cells of these mice. Indeed, an *ex vivo* study found that cultured VSMC challenged with IL-22 did not significantly change inflammatory cytokine mRNA profiles (20). Since IL-22 receptors are found on SMC, but not on hematopoietic cells, IL-22 may be involved in unidirectional T-cell SMC cross-talk (21). In a second study, IL-22 levels were significantly increased in hypertensive patients compared with healthy persons (22). Mice infused with angiotensin II (AII) become hypertensive. The treatment of AII-infused mice with rIL-22 increased the blood pressure, amplified inflammatory responses, and aggravated endothelial dysfunction in these mice. By contrast, infusion with anti-IL-22-neutralizing monoclonal antibody decreased blood pressure, reduced inflammatory responses, and attenuated endothelial dysfunction (22). IL-22 affects smooth muscle cell phenotype and plaque formation in apolipoprotein E knockout mice. While additional studies are necessary to clarify a role for IL-22 in vascular inflammation, it does appear to play a pro-inflammatory, pathological role.

### Interleukin-24

Interleukin-24 was originally identified in healthy melanocytes abut not metastatic melanoma cells and was considered to be a mediator of tumor suppression. IL-24 is predominantly released by activated monocytes, macrophages, and Th lymphocytes in an IL-4-inducible fashion. IL-24 inhibits tumor cell growth by the induction of apoptosis and most attention has been given IL-24 as a cancer therapeutic (23). Very little literature is available concerning a role for IL-24 in vascular pathophysiology; IL-24 does induce the secretion of TNFα and IL-6 from monocytes and therefore would potentially have a pro-atherosclerotic effect *in vivo*. One case–control association study demonstrated the association of IL-24 polymorphisms with metabolic and cardiovascular risk factors in individuals with premature coronary artery disease (CAD) (24). One study using cultured human VSMC suggests that IL-24 inhibits reactive oxygen species (ROS) production, thus reducing ROS-driven VSMC proliferation, a major maladaptive event in atherosclerosis (25). Taken together, IL-24 could have both pro- and anti-atherosclerotic effects, and studies are needed to directly address the role of this cytokine in vascular disease.

### Interleukin-19

**Figure 2:**
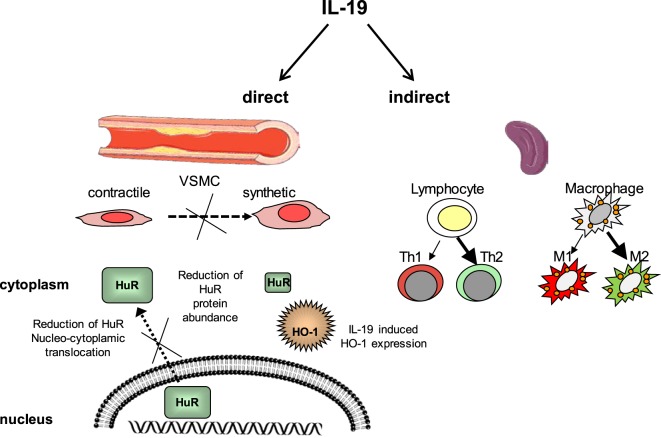
Interleukin-19 (IL-19) has direct and indirect vasculo-protective effects. IL-19 reduces VSMC inflammation and proliferation by reduction of HuR nucleocytoplasmic translocation and reduction of HuR protein abundance. HuR also protects VSMC by the induction of hemeoxygenase-1 expression. IL-19 polarizes T lymphocytes to the Th2 and macrophage to the M2 phenotype, each skewed toward anti-inflammatory and reparative characteristics. Together, this results in less pro-inflammatory and proliferative gene expression, resulting in reduced vascular inflammation. HO-1, Heme oxygenase-1, HuR, Human antigen R, VSMC, vascular smooth muscle cell.

Interleukin-19 was first identified and cloned by searching Expressed Sequence Tag databases for IL-10 homologs (26) and was originally placed in an extended IL-10 family. IL-19 is now considered to be in a subfamily which also includes IL-20, IL-22, and IL-24, which has been alternately referred to as the “IL-19 subfamily” (27) or the “IL-20 subfamily” (28). Although these subfamily members are recognized by and signal through different combinations of shared receptor chain complexes, IL-19 is functionally distinct from these subfamily members and especially IL-10 in terms of cell-specific expression and function. One study suggested that recombinant IL-19 synthesized in the author’s laboratory may have pro-inflammatory effects, in that it induced IL-6 expression; however, no other studies have reported similar effects (29). This same group found that IL-19 was increased in lungs in a murine model of asthma, but also induced the expression of Th2 cytokines in these mice (30). As an anti-inflammatory interleukin, it is not surprising that IL-19 has vasculoprotective effects. We will review IL-19 expression in vascular disease as well as literature suggesting that IL-19 atheroprotective effects are direct, with potent anti-inflammatory effects on resident vascular cells, and also indirect, by polarization of adaptive immunity (Figure 2). These effects are summarized in Table 1.

**Table 1:** Summary of vascular protective effects of interleukin-19 (IL-19).

Vascular protective effect	Species	Source
Expression in diseased vascular tissue	Human and mouse	(31, 35)
Expression in stimulated vascular smooth muscle cell (VSMC) and endothelial cell (EC)	Cultured human cells	(31, 53)
Anti-atherosclerotic	*In vivo* mouse	(35)
Halts preexisting plaque	*In vivo* mouse	(39)
Anti-restenotic	*In vivo* mouse and rat	(31, 41)
Reduces VSMC migration	Cultured human cells	(45)
Antiproliferative	Human and mouse	(31, 41)
Cytoprotective, induces heme oxygenase-1 (HO-1)	Cultured human cells	(48)
Decreases mRNA stability of inflammatory mRNA	Cultured human cells	(35, 39)
Lymphocyte polarization to T helper 2 (Th2)	*In vivo* mouse	(35, 40)
Macrophage polarization to M2	*In vivo* mouse and cultured	(39, 40)
	Human cells	

### Expression of IL-19 in Vascular Disease

Vascular expression of IL-19 was first identified through cDNA microarray analysis, where IL-19 was detected in fetal calf serum-stimulated, but not serum-starved cultured human VSMC (31). This was unexpected as prior to this time, IL-19 expression had assumed to be restricted to leukocytes (14, 32–34). Similarly, immunohistochemical analysis determined that IL-19 protein was undetectable in normal human arteries, but was robustly expressed in neointimal and medial VSMC and leukocytes, and EC in human coronary arteries with allograft vasculopathy, a chronic vascular inflammatory disease (31, 35). Similar results were found in these same cell types in atherosclerotic plaque in aortic arch of ApoE−/− mice, but not aortic arch of wild-type mice. IL-20 receptor subunits are also expressed in these cells (36). Two reports indicate that plasma levels of IL-19 are significantly increased in patients undergoing coronary artery bypass graft surgery and suggest that these increased IL-19 levels contribute to the cell-mediated immune suppression often observed in these patients (37, 38). IL-19 levels were increased in coronary arteries of symptomatic, compared with asymptomatic patients with CAD, suggesting that IL-19 expression may be a compensatory response to inflammatory insult (35).

### IL-19 Is Atheroprotective and Anti-Restenotic

Three *in vivo* studies have shown that IL-19 has atheroprotective effects in mice. In an initial study, LDLR−/− mice fed an atherogenic diet and injected with 1.0 ng/g/day of recombinant IL-19 demonstrated a significantly less atherosclerotic plaque lesion area compared with saline-injected control mice (35). These mice also had reduced macrophage infiltrate into atherosclerotic lesions. Weight gain and serum cholesterol and triglyceride levels were identical in IL-19 and saline control mice, indicating that atheroprotective effects were inflammatory in nature, rather than metabolic. In a second study, LDLR−/− mice, which had preexisting plaque, were injected with rmIL-19 or saline as controls and continued to be fed a high-fat diet for 8 weeks. The injection of 10 ng/g/day rmIL-19 did not reduce the size of the existing plaque, but did completely halt the progression of plaque size compared with saline controls, suggesting that IL-19 may represent a therapy to be used in humans (39). A third study utilized LDLR−/− mice crossed with IL-19−/− mice and fed a HFD. LDLR/IL-19 DKO mice had a significantly increased atherosclerotic plaque size compared with LDLR−/− controls (40). Two *in vivo* studies indicate that IL-19 is potently anti-restenotic. In the first, the delivery of IL-19 adenovirus decreased neointima formation in a rat model of balloon angioplasty-induced restenosis compared to AdGFP controls (31). In a second study, mouse carotid arteries from IL-19−/− mice subject to carotid artery ligation demonstrated a significantly increased media/neointima (N/I) ratio indicative of restenosis, compared with wild-type control mice (41). Importantly, these mice could be “rescued,” in which restenosis could be reduced to wild-type levels, by the injection of IL-19−/− mice with rmIL-19. Together, these five *in vivo* studies suggest that IL-19 confers vascular-protective effects.

### Direct Anti-Inflammatory Effects of IL-19 on Vascular Cells

Several *in vivo* and *ex vivo* studies suggest that IL-19 has potent anti-inflammatory effects on cells outside the immune system. Proliferation of VSMC is a key cellular event in restenosis and allograft vasculopathy. Treatment of cultured VSMC with recombinant IL-19 decreased VSMC proliferation in a dose-dependent manner (31, 41). *In vivo*, the delivery of IL-19 adenovirus not only decreased neointima formation but significantly reduced the number of proliferating VSMC in a rat model of balloon angioplasty-induced restenosis (31). While the addition of, or overexpression of IL-19, is antiproliferative, abrogation of IL-19 results in increased VSMC proliferation (41). Ligation of mouse carotid artery generates a VSMC-rich neointima similar to human models of restenosis (42–44). VSMC explanted from IL-19 KO mice proliferated significantly more rapidly and expressed significantly greater levels of inflammatory cytokine mRNA than wild-type VSMC. This increased proliferation could be inhibited by the addition of IL-19 to cultured KO VSMC.

Medial to intimal VSMC migration is an important component of vascular restenosis. IL-19 inhibited cultured VSMC migration into a scratch wound as well as PDGF-induced chemotaxis in a Boyden chamber (45). This study showed that IL-19 inhibited the activation of cellular motility proteins, including myosin light chain, cofilin, Hsp70, and the monomeric G proteins Rac1 and RhoA. Heme oxygenase-1 (HO-1) is a powerful antioxidant with multiple vasculoprotective effects including the attenuation of VSMC proliferation, a reduction in monocyte arterial transmigration, and a reduction of apoptosis (46, 47). It was found that IL-19 could induce the expression of HO-1 mRNA and protein in cultured VSMC and also reduce peroxide-induced apoptosis and growth-factor-induced ROS accumulation in VSMC (48). This reduction in ROS was abrogated when VSMCs were transfected with HO-1-specific siRNA prior to IL-19 treatment. *In vivo*, IL-19 reduced TNFα-induced ROS accumulation in murine coronary arteries (48), associating IL-19 as a potential link between two vascular-protective systems, anti-inflammation and reduction of ROS.

Many pro-inflammatory and proliferative genes are targeted for the degradation by cis-acting AU-rich elements (AREs) in their 3′ untranslated regions (3′UTR) (49). Human R Antigen (HuR) is a major regulator of ARE-bearing transcripts and thus inflammation, by increasing their mRNA stability (50, 51). A number of studies, in VSMC and EC, demonstrated that IL-19 can decrease the mRNA stability and protein abundance of ARE containing proliferative and pro-inflammatory genes (40, 52, 53). Interestingly, IL-19 was not able to reduce the abundance of proteins which do not contain ARE in their 3'UTR. One presumed means for IL-19 effects is by reduction in HuR abundance, through a yet unidentified mechanism (35, 40). The ability of HuR to stabilize mRNA corresponds with its translocation from a predominately nuclear location into the cytoplasm (50). IL-19 also decreases HuR translocation in both VSMC and EC, in a mechanism that remains to be elucidated (52, 53). Other Th2 interleukins can reduce inflammatory cytokine expression, primarily by the inhibition of activation of the transcription factor NF-κB. In contrast to these interleukins, IL-19 does not inhibit NF-κB activation (52, 53). In this fashion, IL-19 produces a posttranscriptional decrease in the abundance of these transcripts through the inhibition of HuR abundance and translocation, thereby mediating a decrease in pro-inflammatory transcript stability. Future studies are needed to identify the precise mechanisms by which IL-19 decreases HuR abundance and translocation.

Together, antiproliferative, anti-migratory, anti-ROS-generating, and a decrease in inflammatory mRNA stability provide the molecular mechanisms for IL-19 atheroprotective and anti-restenotic activity. The larger picture suggests that the vascular expression of IL-19 in response to injury might represent a novel autocrine or paracrine mechanism for attenuation and regulation of vascular inflammation (Figure 1).

### IL-19 Polarizes Adaptive Immunity to an Anti-Inflammatory Phenotype

Interleukin-19 is considered to be a Th2 interleukin because it promotes the Th2, rather than the Th1 response in lymphocytes, although the mechanism which drives this response has not yet been fully characterized (14, 27, 33). In vascular inflammatory diseases, where the participating cell types include immune cells as well as vascular cells, the attenuation of global inflammatory responses also reduces the severity of vascular disease. A number of studies suggest that IL-19 polarizes adaptive immunity by promoting Th2 responses in a positive feedback loop increasing IL-4-positive and less IFNγ-positive leukocytes (30, 32, 33). LDLR−/− mice fed an atherogenic HFD diet are polarized to Th1 since hyperlipidemia is pro-inflammatory (11, 54). In the context of vascular protection, three studies indicate that IL-19 polarizes adaptive immunity in response to atherogenic stimuli. In one study, when LDLR−/− mice received daily injections of rmIL-19, they express less Th1 markers (IFNγ, IL-12, T-bet, and TNFα) and greater expression of Th2-associated markers such as GATA3 and FoxP3 (35). In a second study using LDLR−/− mice fed a HFD, daily rmIL-19 injection promoted the activation of key pathways leading to M2 macrophage polarization, including STAT3, STAT6, and increased expression of Kruppel-like factor 4, and peroxisome proliferator-activated receptor γ (39). A third study indicated that in LDLR/IL-19 DKO, T cells express significantly greater Th1 markers, and macrophage expresses greater amounts of M2 markers in both plaque and spleen (40). Taken together with previous studies, this suggests both local and global polarization of adaptive immunity by IL-19 (Figure 1).

### Summary

The culmination of work on the IL-20 family cytokines indicates a wide variety of source and target cells, suggesting a plethora of potential roles of these cytokines in numerous pathologies. Interleukin-19, in particular, effects both vascular and inflammatory cells, providing dual-pronged therapeutic potential to combat vascular inflammatory syndromes. Future studies are necessary to determine the precise molecular and cellular mechanisms for IL-19-mediated decreases in vascular disease. For example, which is the primary atheroprotective mechanism: reduction of VSMC inflammatory gene expression or polarization of adaptive immunity? A body of work is emerging which implicates IL-19 as a previously unrecognized, counterregulatory, protective factor for numerous vascular diseases.

## Author Contributions

The author confirms being the sole contributor of this work and approved it for publication.

## Conflict of Interest Statement

The author declares that the research was conducted in the absence of any commercial or financial relationships that could be construed as a potential conflict of interest.
